# Multimodality Imaging in the Evaluation and Prognostication of Cardiac Amyloidosis

**DOI:** 10.3389/fcvm.2022.787618

**Published:** 2022-03-24

**Authors:** Paul J. Scheel, Monica Mukherjee, Allison G. Hays, Joban Vaishnav

**Affiliations:** Division of Cardiology, Johns Hopkins University School of Medicine, Baltimore, MD, United States

**Keywords:** cardiac amyloidosis, echocardiography, cardiac magnetic resonance imaging (CMR), endomyocardial biopsy, cardiac scintigraphy, nuclear imaging

## Abstract

Cardiac amyloidosis (CA) is an infiltrative cardiomyopathy resulting from deposition of misfolded immunoglobulin light chains (AL-CA) or transthyretin (ATTR-CA) proteins in the myocardium. Survival varies between the different subtypes of amyloidosis and degree of cardiac involvement, but accurate diagnosis is essential to ensure initiation of therapeutic interventions that may slow or potentially prevent morbidity and mortality in these patients. As there are now effective treatment options for CA, identifying underlying disease pathogenesis is crucial and can be guided by multimodality imaging techniques such as echocardiography, magnetic resonance imaging, and nuclear scanning modalities. However, as use of cardiac imaging is becoming more widespread, understanding optimal applications and potential shortcomings is increasingly important. Additionally, certain imaging modalities can provide prognostic information and may affect treatment planning. In patients whom imaging remains non-diagnostic, tissue biopsy, specifically endomyocardial biopsy, continues to play an essential role and can facilitate accurate and timely diagnosis such that appropriate treatment can be started. In this review, we examine the multimodality imaging approach to the diagnosis of CA with particular emphasis on the prognostic utility and limitations of each imaging modality. We also discuss how imaging can guide the decision to pursue tissue biopsy for timely diagnosis of CA.

## Introduction

Systemic amyloidosis is terminology for a broad spectrum of diseases that result from the aggregation of misfolded proteins. Cardiac amyloidosis (CA) occurs when amyloid fibrils accumulate in the myocardium often resulting in a restrictive cardiomyopathy (1–3). The two most common proteins that lead to CA include monoclonal immunoglobulin light chains (AL-CA) and transthyretin (ATTR-CA). AL-CA is rare with an incidence of 10–12 per million person-years and a slight male predominance (4). ATTR-CA, on the other hand, has a significant male predominance and is likely more common than reported and as such, a true estimate of prevalence is difficult (2). An autopsy study of individuals greater than 80 years old found ATTR-CA in 25% of subjects, though only a subset of those were thought to be clinically relevant (5). The misfolded amyloid fibrils in ATTR-CA can either occur due to an age-related phenomenon, known as wild-type CA (wtATTR), or related to a genetic variant, known as hereditary CA (hATTR). There are more than 130 TTR mutations identified, with the most common mutation in the United States being Val122Ile, present in 3–4% of African Americans (6). While the amyloid fibril deposition in AL and ATTR-CA can be systemic (it is the cardiac involvement that determines prognosis). Survival for treated AL-CA has improved with advances in chemotherapy (7). ATTR-CA survival varies between subtypes, but in general is 3–5 years without treatment (2). With the advancements in the treatment of both AL and ATTR-CA, there is a need for increased recognition of this condition. Additionally, the efficacy of available therapies for CA is far more favorable if instituted earlier in the disease course, highlighting the need for early diagnosis and treatment.

Although direct tissue characterization with organ biopsy had historically been the mainstay for diagnosis of CA, more recently non-invasive cardiac imaging algorithms have become the cornerstone of evaluation (8, 9). These algorithms are typically predicated on establishing suspicion for CA based on clinical factors and complementing that with imaging findings. If imaging affirms clinical suspicion, laboratory evaluation, and nuclear techniques or tissue biopsy are needed to determine specific amyloidosis subtype (8–10). However, the test performance characteristics and prognostic utility are evolving given more widespread application and treatment. In this review, we examine the multimodality imaging approach to the diagnosis of CA. We highlight the evolution in diagnostic performance, complementary information, and prognostic utility of each modality as well as emerging imaging techniques in the diagnosis and management of CA. Finally, we focus on how multimodality imaging can guide clinicians on when to pursue tissue biopsy in select cases.

## Clinical Recognition of Cardiac Amyloidosis

Cardiac amyloidosis is under-recognized and the diagnosis often delayed, with the majority of patients experiencing greater than 10 healthcare interactions in the 3 years before accurate diagnosis (2). CA should be suspected in any patient presenting with heart failure (HF), a non-dilated left ventricle (LV), and unexplained left ventricular hypertrophy (LVH). However, as disease prevalence may increase with age, particularly for wtATTR, patients may have co-morbid conditions such as hypertension, ischemic heart disease, or aortic stenosis that confound the diagnosis. Therefore, it is important to maintain a high index of suspicion and also screen for other cardiac clues that may suggest underlying ATTR-CA as the driver of HF (Table 1). Additionally, there are non-cardiac manifestations of CA that may precede overt cardiac symptoms and recognition of such clues may allow for an earlier diagnosis of ATTR-CA (Table 1). AL amyloidosis is a systemic condition, and may present with proteinuria, macroglossia, periorbital purpura, or neuropathy. Once AL-CA is suspected, clinicians should aim to confirm the diagnosis within 1–2 weeks, as AL-CA should be considered a diagnostic emergency due to the rapid progression of disease without treatment.

**TABLE 1:** Cardiac and non-cardiac clinical clues for possible ATTR-CA.

Non-imaging cardiac red flags for ATTR-CA	Non-cardiac red flags for ATTR-CA
Intolerance of GDMT	Polyneuropathy
Persistent low-level troponin elevation	Autonomic dysfunction/Orthostatic hypotension
Unexplained AV block	Bilateral carpal tunnel syndrome
Family history of cardiomyopathy	Lumbar spine stenosis
HFpEF diagnosis in the absence of risk factors	Diarrhea alternating with constipation

*GDMT, guideline directed medical therapy for heart failure; HFpEF, heart failure with preserved ejection fraction.*

## Echocardiography

**FIGURE 1:**
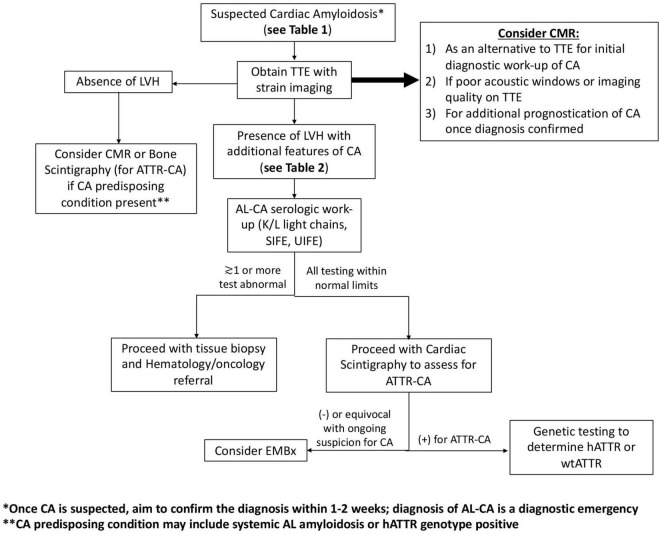
Proposed algorithm for diagnosis of cardiac amyloidosis. Algorithm for patients being assessed for cardiac amyloidosis based on heart failure or risk factors for AL or ATTR-CA. EMBx, endomyocardial biopsy; CMR, cardiac magnetic resonance imaging; LVH, left ventricular hypertrophy; SIFE, serum protein electrophoresis with immunofixation; TTE, transthoracic echocardiogram; UIFE, urine protein electrophoresis with immunofixation.

**FIGURE 2:**
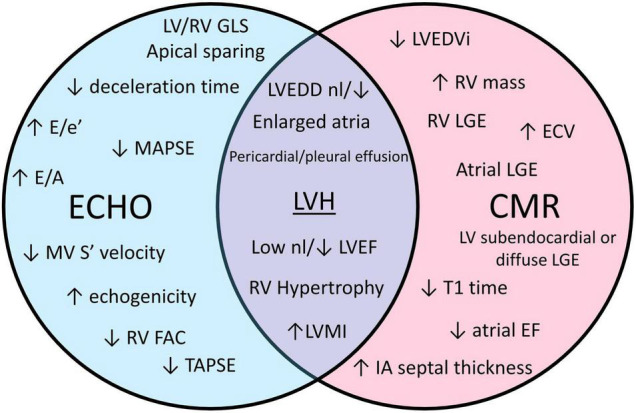
A Venn diagram comparing echocardiogram (echo) and cardiac magnetic resonance (CMR) characteristics seen in cardiac amyloidosis. ECV, extra cellular volume; EF, ejection fraction; FAC, fractional area change; GLS, global longitudinal strain; LGE, late gadolinium enhancement; LV, left ventricle; LVEDD, left ventricular end diastolic diameter; LVEDVi, left ventricular end diastolic volume index; LVH, left ventricular hypertrophy; LVMI, left ventricle mass index; MAPSE, mitral annular plane systolic excursion; MV, mitral valve; nl, normal; RV, right ventricle; TAPSE, tricuspid annular plane systolic excursion.

Transthoracic echocardiogram (TTE) is a mainstay of initial HF evaluation and a common imaging modality that will raise initial diagnostic suspicion of CA (Table 2). Patients undergoing echocardiography eventually diagnosed with CA will likely fall into two categories: (1) symptomatic, with clinical clues to suggest underlying CA as part of evaluation for congestive HF or (2) asymptomatic but at risk for CA because of known non-cardiac AL amyloid involvement or family history of hATTR with known genotype positivity (Figure 1). While TTE is a widely available and helpful diagnostic test that may raise the initial suspicion for CA, demonstration of tissue uptake of the specific type of abnormal protein, either with advanced imaging or biopsy, will always be needed to confirm a diagnosis of CA. Abnormal parameters commonly seen on echocardiography are summarized in Figure 2.

**TABLE 2:** Comparison of each imaging modality with their specific findings in cardiac amyloidosis as well as their relative strengths and weakness.

Imaging modality	Findings in cardiac amyloidosis	Strengths	Limitations
Echocardiography	LVH	Readily available	No differentiation between CA subtypes
	Small LV cavity	Cheap	Variable image quality
	Large atria	High temporal resolution	Early findings in CA non-specific
	RV/LV systolic dysfunction	Identify other causes of LVH (AS, HCM, etc.)	
	Abnormal LV diastolic function	No radiation	
	Abnormal strain	Patient ease	
	Pericardial/pleural effusion		
Magnetic resonance imaging (MRI)	Similar morphologic findings to echocardiography (Figure 2)	Reproducible	Expensive
	Late gadolinium enhancement in atria and ventricles	Direct tissue characterization	Limited availability
	Pericardial/pleural effusion	No radiation	Special expertise required
	Atria dysfunction	Identify other causes of LVH (HCM, infiltrating disease)	Multiple patient specific exclusions (implants, claustrophobia, etc.)
	Interatrial septum thickening	Higher spatial resolution and multi-dimensional strain	
	Abnormal strain		
Cardiac scintigraphy (PYP, DPD, and HDMP)	Increased radiotracer uptake	Cheap	Radiation
	Increased H/CL ratio	Widely available	Mostly qualitative
		Ease of interpretation	Genetic variant uptake variability
		Differentiate amyloid subtype	
PET imaging	Increased radiotracer uptake	Quantitative assessment	Radiation
		Differentiate amyloid subtype	Expensive

*AS, aortic stenosis; H/CL, heart/contralateral; HCM, hypertrophic cardiomyopathy; LV, left ventricle; LVH, left ventricular hypertrophy; PET, positron-emission tomography.*

### Morphologic Changes

**FIGURE 3:**
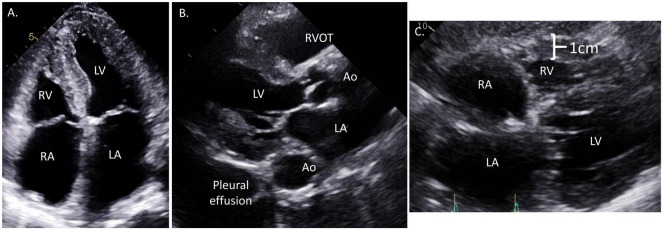
Representative echocardiographic images from a patient with cardiac amyloidosis. **(A)** Apical 4-chamber view showing moderate concentric left ventricular hypertrophy with more prominent proximal septal hypertrophy, significant bi-atrial enlargement, and diffusely thickened atrioventricular valves. **(B)** Parasternal long axis view showing large pleural effusion, moderate concentric hypertrophy, small left ventricular end diastolic diameter (LVEDD), and right ventricle outflow track dilation. **(C)** Subcostal view demonstrating significant right ventricular hypertrophy, biatrial enlargement, and interatrial septal thickening.

LVH is the hallmark finding of CA on echocardiography and is generally considered a prerequisite to pursuing further investigation for CA in patients not otherwise at risk (Figures 3A,B). LVH is defined as septal or posterior LV wall thickness greater than 1.1 cm in men and greater than 1.0 cm in women (11). LVH represents either increased muscle mass (true hypertrophy) or increased presence of a non-muscle substance like amyloid fibrils (pseudohypertrophy). LVH in CA is typically concentric and symmetric but asymmetric hypertrophy can also be seen (12, 13). A normal wall thickness in a patient with known AL amyloidosis traditionally signified a lack of cardiac involvement but very early involvement of disease is also possible in this circumstance (14, 15).

Typically, LVH in ATTR-CA is slowly progressive, allowing for early detection of disease prior to significant symptom onset. In a study by Itzhaki Ben Zadok et al., echocardiograms in AL and ATTR-CA patients analyzed in the years prior to a formal diagnosis found LVH ≥ 12 mm in 79% of patients more than 3 years prior to diagnosis (16). Additionally, ATTR-CA patients developed LVH earlier than AL-CA patients prior to their formal diagnosis, possibly related to the rapid disease progression seen with AL-CA as compared to ATTR-CA (12). LVH, especially mild LVH (wall thickness < 1.5 cm), can have many causes including hypertensive heart disease (HHD), hypertrophic cardiomyopathy (HCM), aortic stenosis, or other infiltrative cardiomyopathies but if additional clinical or morphologic features consistent with CA are present (Table 1), further testing for CA should be pursued. Characteristic echocardiographic findings combined with epidemiologic and clinical clues frequently differentiates between other causes of LVH but overlap can still exist and may requiring additional diagnostics, such as cardiac magnetic resonance imaging (CMR) or genetic testing, to aid diagnosis (Table 3). Of note, low voltage on electrocardiogram (ECG) especially in the setting of LVH on imaging was once considered a hallmark sign of CA but many studies demonstrate this only occurs in a minority of CA patients and may be a finding suggestive of advanced disease (17–19). Sensitivity of low voltage on ECG for CA can be increased by using a Sokolow-Lyon index of ≤ 15 mm (sum of S wave in V1 plus R wave in either V5 or V6, whichever is larger; >35 mm indicates true LV hypertrophy) or combining objective measures of ECG voltage and LV mass based on imaging (17, 20).

While LVH is universal in the current diagnostic paradigm for CA, other morphologic changes occur with variable frequencies in CA (Figure 2). LV cavity size is generally normal or decreased (15, 21), except in the rare circumstance where CA develops after development of an unrelated dilated cardiomyopathy. Compared to other causes of LVH, a small LV cavity size may point more toward CA (7). LV mass index (LVMI), a specific measure of LVH utilizing LV wall thickness and cavity dimension, also tends to be higher in CA compared to other causes of LVH (18). More so, right ventricular hypertrophy (RVH) and significant left atrial enlargement (left atrial volume index, LAVI ≥ 47 mL/m^2^) may be a more specific sign for CA in those patients with LVH (18, 22, 23). Thickened cardiac valve leaflets related to amyloid deposition have been observed in some patients, but is subjective given lack of defined parameters. Pericardial or pleural effusion is also common, and has been described in up to 50% of patients with CA (24, 25).

Many studies initially described increased echogenicity or “sparkling” of the myocardium on TTE in patients with CA (25–28). Advances in image processing has made this feature less pronounced. Contemporary studies have also shown that this is fairly subjective between readers and less common than previously reported (18).

### Diastolic Function

**FIGURE 4:**
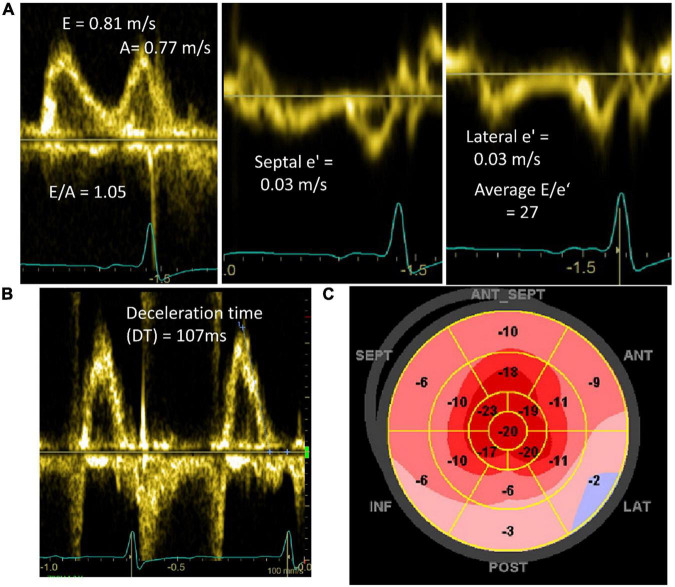
Doppler and longitudinal strain abnormalities in patients diagnosed with cardiac amyloidosis. **(A)** Mitral inflow and mitral annular tissue Doppler showing pseudonormal diastolic filling (Grade 2) and high E/e′ signaling elevated left atrial pressures. **(B)** Mitral inflow pulse wave Doppler of patient in atrial fibrillation demonstrating short deceleration time (DT) (normal 130–220 ms). **(C)** Peak systolic longitudinal strain map demonstrating reduced longitudinal strain in basal and mid ventricular segments with relative apical sparing.

Aberrations in Doppler based diastolic parameters in CA was recognized soon after this technology was developed (29). These abnormal parameters include shorter deceleration time (DT), higher E/A, and higher E/e′ (Figure 4). A limitation for diastolic assessment in CA is atrial fibrillation, a common comorbidity in this condition (30). CA typically leads to more severe diastolic dysfunction compared to other causes of LVH (18, 21, 23) and diastolic parameters may worsen with disease progression (10, 15, 31). Chacko et al. (32) showed that a spectrum of diastolic function exists even within ATTR-CA and depending on the underlying genetic variant, if present. Despite having similar wall thickness, wtATTR patients had worse diastolic function compared to Thr60Ala hATTR patients, though both had better diastolic function than Val122Ile hATTR patients (32). The variations in diastolic function based on genetic subtype correlated with differences in symptoms, biomarkers, and mortality (32).

### Systolic Function

Early models classified CA as a “diastolic heart failure” with systolic dysfunction being a later manifestation of disease (15). However, even early CA may have subtle abnormalities in systolic function. Left ventricular ejection fraction (LVEF) in CA can be normal or low-normal, though typically lower as compared to other causes of LVH (18, 21–23). Mitral annular plane systolic excursion (MAPSE) is frequently abnormal in CA especially as HF develops but this is not performed on routine TTEs and may be a late manifestation of disease (33). Tissue Doppler measuring mitral annular velocity (S′) is another marker of systolic function and a cutoff ≤ 6 cm/s performed well as a screening test for possible CA in a population of patients with severe aortic stenosis (34). The presence of RV systolic dysfunction in CA, which may occur due to direct amyloid infiltration or related to left heart disease in CA, is currently an area of active investigation. Both AL and ATTR-CA patients have been shown to have lower RV fractional shortening and tricuspid annular plane systolic excursion (TAPSE) compared to patients with alternative causes of LVH (35, 36). Licordari et al. examined 37 patients with a known pathogenic TTR variant with no or minimal symptoms and found that in those with CA, echocardiographic markers of RV function were already impaired in this early stage of disease (37). RV dilation is non-specific and can be seen later in the disease course as well (38).

**TABLE 3:** Clinical and echocardiographic characteristics of alternative causes of left ventricular hypertrophy beyond cardiac amyloidosis.

Hypertrophic disease	Clinical clues	Echocardiographic clues
Fabry disease	Childhood to adulthood	Concentric/symmetric LVH
	X-linked inheritance	Papillary hypertrophy
	Abdominal pain	Papillary position variants
	Angiokeratomas	RV hypertrophy
	Kidney abnormalities	Regional strain abnormalities
	Hypohidrosis	Occasional areas of LV thinning
	Conduction disease	
Hypertrophic cardiomyopathy	Childhood to adulthood	Most commonly asymmetric LVH
	Sudden cardiac death	Apical/mid-cavity variants
	Often inherited	Apical aneurysm
	ECG pattern often	Papillary position variants
	characteristic	MV abnormalities
		Mitral regurgitation
Hypertensive heart disease (HHD)	Long-standing documented HTN	Non-specific
	Strong family history HTN	
	Multiple anti-hypertensives	
Athlete’s heart	Endurance/high-intensity exercise	Eccentric hypertrophy (LV dilation)
	Resting bradycardia	RV dilation/hypertrophy
		Atrial dilation
		Non-compaction sometimes seen
Friedreich ataxia	Usually childhood/adolescent	Asymmetric or concentric LVH
	Autosomal recessive	Dilated variants
	Gait ataxia	
	Vision/hearing problems	
	Nystagmus	
Danon disease	Childhood/adolescent	Non-compaction
	X-linked inheritance	
	Skeletal myopathy	
	Cognitive impairment	
	Pre-excitation on ECG	
Left ventricular non-compaction (LVNC)	Childhood to adulthood	Non-compaction
	Thromboembolic events	Colour Doppler of hypertrophy for flow
	Malignant arrhythmias	Contrast usage may help
Hypereosinophilic cardiomyopathy	Hypereosinophilic state	Restrictive
	Thromboembolic events	Endomyocardial thickening
	Fulminant to indolent	LV/RV thrombi
		MV/TV entrapment
Mitochondrial cardiomyopathies	Childhood	Non-compaction
	Often maternal inheritance	
	Multiple distinct syndromes	
	Myopathy	
	Stressors worsen symptoms	
RASopathies	Childhood	Other heart defects
(Noonan, etc.)	Myopathy	
	Developmental delay	
	Often characteristic appearance	

*HTN, hypertension; LV, left ventricle; LVH, left ventricular hypertrophy; MV, mitral valve; RV, right ventricle; TV, tricuspid valve.*

### Strain Imaging

Left ventricular strain imaging using speckle tracking echocardiographic techniques measures the regional and global deformation of the myocardium (19, 39). The reduction in strain and strain rate occurs before overt myocardial dysfunction allowing for earlier detection of systolic dysfunction (14). Initial studies of strain in CA demonstrated clear differences between patients without CA and those with CA even in the absence of overt HF (15). Furthermore, CA patients with HF have worse strain compared to those who were asymptomatic, suggesting that abnormal strain may occur as a continuum related to disease severity (15).

As strain imaging was increasingly used in CA, a distinct pattern of “relative apical sparing,” referring to a reduced longitudinal strain (LS) rate in the basal to mid-ventricular segments of the LV compared to the apical segments, was recognized (Figure 3C). While strain was already shown to be reduced in CA compared to other causes of LVH (21), this pattern may be of additive diagnostic value for CA. Relative apical longitudinal strain (RALS; = [average apical LS]/[average basal LS + average mid LS]) is an objective measure of the apical sparing pattern. Using a cutoff off of RALS > 1, CA could be differentiated from other causes of LVH with a sensitivity > 90% and specificity >80% in 55 AL and ATTR-CA patients (19). However, a slightly larger study that included AL-CA, wtATTR, and hATTR demonstrated apical sparing in a minority (48%) of patients (39). This may be due to early studies on LS in CA including predominantly AL-CA patients since this phenomenon is less common in ATTR-CA (19). Another explanation for these differences is that apical sparing may vary based on disease stage. Therefore, while an apical sparing pattern can certainly be seen in CA and may enhance the likelihood of diagnosis, the absence of this pattern should not eliminate the possibility of a CA diagnosis. Additional novel echocardiographic markers have been derived from strain imaging to help differentiate CA from other causes of LVH including the LVEF to global longitudinal strain (GLS) ratio (EFSR). A study comparing CA to HHD and HCM patients showed an EFSR > 4.1 had sensitivity and specificity of 90% for detecting CA in this population (18).

Right ventricular strain can also be measured on echocardiogram; however, this technique is not used in widespread clinical practice. Palomero and colleagues (40) evaluated RV strain in a group of AL and ATTR-CA patients (n = 78) compared to controls. All patients had reduced biventricular function and LV apical sparing. Notably an RV apical sparing pattern was only seen in AL-CA patients. AL-CA patients also had worse RV function measured by traditional parameters, which could support worsening RV function existing on a continuum of disease severity (40).

In addition to the atrial morphologic changes that can be seen as a secondary hemodynamic consequence from any cause of ventricular impairment, there is increasing evidence to suggest CA can also impair atrial function as measured by atrial strain. Diagnostically, left atrial strain can help differentiate CA from other causes of LVH (41–43). Multiple studies have demonstrated significant reduction in left atrial strain parameters in both AL and ATTR-CA compared to HHD despite similar degrees of LV wall thickness. Left atrial strain outperformed LV RALS in discriminating between disease states (41, 43). Another study also showed that while both ATTR-CA and HCM patients have reduced peak LA strain compared to controls, the degree of reduction was greater in ATTR-CA patients providing another potential discriminatory variable (42). Atrial strain correlates with other echocardiographic markers of disease burden (41). Furthermore, both LA and right atrial (RA) strain have prognostic value for CA patients and independently predict mortality (44). Atrial strain techniques are mostly isolated to research protocols but with a growing body of evidence demonstrating diagnostic and prognostic utility, clinical application will likely increase in the coming years.

### Multi-Parametric Scores

The multitude of abnormal echocardiographic parameters seen in CA has led to the development of multi-parametric scores for the diagnosis of CA. These may be particular importance in early disease when the echocardiographic changes are subtle and less specific (10, 45). Boldrini et al. (22) studied >1,000 patients who either had proven systemic AL amyloidosis or LVH suspicious for possible CA who then underwent subsequent work-up with myocardial or non-myocardial biopsy. Patients with suspected ATTR-CA underwent bone scintigraphy and 85% of patients also underwent CMR. For patients with AL amyloidosis, there was higher specificity for cardiac involvement, with more points using relative wall thickness (RWT = 2 × PWd/LVEDD, >0.52, two points), E/e′ (>10, two points), TAPSE (≤19 mm, one point), and LS (≥−14%, one point). For patients referred with LVH and possible CA, more points increased specificity for CA using RWT (>0.6, three points), E/e′ (>11, one point), TAPSE (≤19 mm, two points), LS (≥−13%, one points), and septal longitudinal systolic apex-to-base ratio (SAB, >2.9, three points). SAB is a measure similar to RALS as a measure of apical sparing (22).

Aimo and colleagues developed a simpler echocardiographic score to maximize specificity of the diagnosis. The Amyloidosis Index (AMYLI) score equals RWT × E/e′, with its main limitation being the exclusion of patients in atrial fibrillation during echocardiogram. A cutoff < 2.36 in patients with systemic AL amyloidosis and <2.22 in unexplained LVH excluded CA (22). The AMYLI score was compared to the two scoring systems developed by Boldrini et al. and demonstrated non-inferiority for the exclusion of CA (23). CA is primed for the use of multi-parametric echocardiography scores and we suspect the applicability of such scores will continue to increase. These scores help to objectify the echocardiographic parameters many clinicians may notice by clinical gestalt but can be overlooked in combination. More widespread use could decrease delays in diagnosis and treatment.

### Prognosis

The prognostic significance of echocardiographic findings in CA has been long recognized (31). In early studies, the most useful echocardiogram predictor of mortality was E/A closely followed by shorter DT and lower fractional shortening (15, 31). These same parameters correlate with HF severity and mortality. Contemporary studies added apical LS and lower LVEF as an independent predictor of major adverse cardiac events (MACE) in patients with CA (39). RV function in CA has also been shown to predict mortality with lower TAPSE and RV strain correlating with MACE in some studies (36). A recent study consisting predominantly of AL-CA patients demonstrated the prognostic significance of both RA and LA strain (44). It remains to be seen how these prognostic echocardiographic measures are affected by CA treatment and specifically if these parameters may be helpful in predicting treatment response.

### Emerging Techniques

Despite increasing knowledge about numerous echocardiographic changes in CA, final diagnosis is contingent on further testing based on level of suspicion, which may vary from clinician to clinician. Multiparametric scores can help streamline this diagnostic assessment, but may not be readily applied in clinical practice, and still relies on maintaining an underlying diagnostic suspicion for CA (2). Machine learning (ML) provides the potential to bridge this important diagnostic pitfall. ML methodology refers to a computational approach that incorporates a multitude of complex data structures to agnostically identify relationships commonly seen in disease patterns without explicit instruction. The variable and diverse echocardiographic changes seen in CA, even in early disease, could potentially be detected by ML and signal to clinicians to consider further testing. Goto et al. (46) performed a large multicenter study using ML techniques of both ECG and echocardiograms in CA. They used a video-based model for echocardiography using a single apical 4-chamber view. Their model was able to detect CA by echocardiography with a C-statistic ≥ 0.85 at each site up to 1 year prior to diagnosis. Additionally, their model was able to discriminate CA from other diseases causing LVH including HCM, HHD, and end-stage renal disease with C-statistic ≥ 0.90 at each site. Their model outperformed two expert echocardiography readers in diagnostic accuracy. The area under the curve (AUC) for differentiating between causes of LVH ranged between 0.87 and 0.96 at each institution which is comparable or higher than the AUC for similar populations using the multiparametric scores developed by Boldrini and Aimo (22, 23). The ECG model also performed well on its own across sites. When combining ECG and echocardiography in a step-wise fashion, they demonstrated a positive predictive value (PPV) of nearly 75% across two sites (46). A layered testing and referral algorithm using ML on initial ECG and subsequent echocardiography holds promise in detecting more CA cases and earlier in the disease course. However, as with all testing modalities, the impact on performance with more widespread use will need to be examined.

## Magnetic Resonance Imaging

Magnetic resonance imaging (MRI) utilizes strong magnetic fields in different orientations to excite hydrogen atoms then measures the emitted signal as they relax. Hydrogen atoms in different tissues have distinct excitation and relaxation properties based on their surrounding structure making it possible to characterize tissue properties. CMR was first employed over 3 decades ago and has been used for diagnosis and prognostication with increasing frequency in multiple cardiac conditions. Given the high chamber fidelity, it is considered the gold standard for chamber size quantification and EF measurement. CMR is less subject to limitations in image quality compared to echocardiogram, but requires a higher level of expertise, is costlier, and is not as widely available (Table 2). The main patient-related limitations to CMR are body habitus, claustrophobia, non-MRI compatible metallic implants, and severe renal dysfunction if gadolinium-based contrast agents are to be used.

**FIGURE 5:**
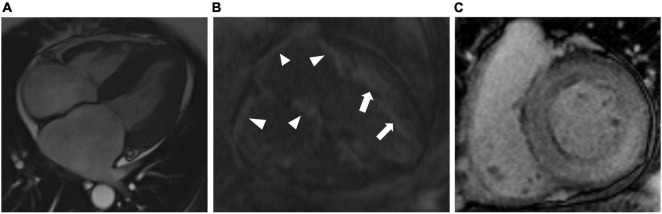
Representative cardiac magnetic resonance images (CMR) from patients with cardiac amyloidosis. **(A)** T2 TRUFI 4-chamber view showing biventricular hypertrophy, biatrial enlargement, and pericardial effusion. **(B)** T1 4-chamber view late post-contrast image in a patient with ATTR-CA showing diffuse atrial LGE (arrowheads) and left ventricular subendocardial LGE (arrows). **(C)** T1 short-axis late post-contrast image in a patient with AL-CA showing diffuse LGE of the left ventricle.

Cardiac magnetic resonance may be utilized in the initial evaluation of CA or to supplement initial diagnostic suspicion of CA based on echocardiogram in select patients (Figure 1). Acquisition of CMR should ideally not delay diagnosis of CA, as additional modalities are typically necessary to confirm a diagnosis of CA prior to initiating treatment. CMR is excellent at demonstrating morphologic changes in CA, similar to echocardiogram and therefore, is very beneficial in patients with poor acoustic windows on echocardiogram. CMR adds significant information on tissue characterization of the myocardium compared to echocardiogram (Figure 2), which is enhanced with the use of gadolinium-based contrast agents. Additionally, improved tagging and strain techniques in CMR allow for more detailed myocardial deformation analysis than currently provided by echocardiography. CMR technology is continually improving as novel techniques and applications are being developed. Representative CMR images for a patient with CA are shown in Figure 5.

### Morphologic Changes

As with echocardiogram, LVH is the hallmark finding of CA on CMR regardless of the underlying type of CA. In studies comparing findings on CMR in patients with CA to those of healthy volunteers, patients with CA had higher LVMI, lower LV end diastolic volume index (LVEDVI), and lower LVEF (47). However, in one study of 36 patients with HF and either myocardial biopsy or autopsy evidence of CA (n = 11) or extra cardiac biopsy plus LVH on echocardiogram, nearly one-third of patients had normal LVMI by CMR (47). This suggests that CA may be present to some extent without frank hypertrophy as measured by LVMI on CMR. There may also be differences in the degree of LVH on CMR between the types of CA, with wtATTR generally having higher LVMI, possibly related to the elderly demographic and longer asymptomatic disease state compared to AL-CA (48). CMR can help differentiate between CA and other diseases that cause LVH. Specifically, compared to HHD controls, patients with CA have been shown to have lower LVEF and right ventricular ejection fraction (RVEF), higher LVMI, higher RV mass, and greater degree of RVH on CMR (49–52). Differences in degree of RVH may also exist between types of CA, occurring less commonly in AL-CA (53).

While both right and left ventricular changes can be seen on echocardiogram, CMR outperforms echocardiogram at assessment of atrial abnormalities. Increased interatrial (IA) septum thickness originally had promise as a fairly specific finding in CA compared to other causes of LVH (49, 50, 54). Proposed IA thickness cutoffs ranged from greater than 5 to 6 mm. Contemporary studies do not show this finding with as high of frequency possibly because of earlier CA diagnosis (47). Additionally, there may be differences in frequency of increased IA thickness based on type of CA, with this being more common in wtATTR (48). Kwong and colleagues recently demonstrated lower total LA emptying in the setting of higher LA volumes in CA compared to those with HHD and non-ischemic cardiomyopathy (55). This, in addition to other LA parameters, had high likelihood ratios in differentiating CA from other disease states. Given the unique atrial abnormalities that may be present in CA, imaging of the atria should be given special attention in patients undergoing evaluation for CA.

Echocardiography, as compared to CMR, remains the preferred modality for examination of diastolic filling patterns. Rubinshtein et al. (56) compared diastolic function in patients with CA utilizing echocardiography and CMR. In general, E/A was lower by CMR compared to echocardiography but DT correlated well across both imaging modalities. However, there was a subset of patients in whom diastolic dysfunction assessment significantly differed across the imaging modalities, with the more severe pattern identified by echocardiogram (56). Without significant leaps in CMR technology and given the improvements in time resolution on echocardiogram, echocardiogram will likely remain as the preferred modality for assessment of diastolic dysfunction in CA and other disease states.

### Tissue Characterization

The major strength of CMR as compared to echocardiogram in evaluation of CA lies in the superior detection of myocardial tissue properties. A typical CMR sequence for CA will start with cine images in various orientations to assess function and other static measurements. After these are obtained, most of the remaining sequences are designed to assess various properties of tissue. Amyloid fibril deposition in the myocardium alters the myocardial tissue and therefore CMR characteristics such as T1 relaxation and T2 decay. T1 relaxation varies between tissues based on the time it takes for hydrogen atom spin to reorient in the direction of applied magnetic field after it was altered with a perpendicular radiofrequency pulsation. In the heart, fat and gadolinium (Gd), if used, have short T1 and will appear bright in these sequences. Tissue T2 decay is based on the time it takes for a hydrogen atom to spin out of phase. Fat and water have long T2 and will appear bright on these sequences. The addition of Gd based contrast agents enhances tissue characterization. Traditionally, Gd contrast was used to detect areas of increased extracellular space that may be caused by fibrosis or ischemic scar. These areas had delayed wash-out of contrast, known as late gadolinium enhancement (LGE). In CA, this likely works in a similar manner, but there also appears to be altered Gd kinetics caused by the amyloid fibrils (50). However, the early work on CMR in CA was marked by inconsistencies leading to disparate conclusions for LGE assessment. Additionally, there are a number of operator-dependent factors in CMR imaging such as amount of time delay from injection to imaging and the selection of time intervals based on initial images. Over time, more standardized CMR imaging protocols were developed for CA that have allowed for more uniform interpretation of the results.

The extracellular localization of Gd-based contrast agents made contrast-enhanced CMR the initial technique of interest in CA. Early (<5 mins) after contrast injection, patients affected by CA frequently (60–90%) have abnormal enhancement of the myocardium (47, 50). Maceira et al. (50) noted shorter T1 times of the myocardium with a cut-off of 535 ms having a sensitivity and specificity of 86% and 75%, respectively, for distinguishing CA from HHD controls. However, they also noticed increased contrast clearance from the blood pool leading to higher T1 times. By taking the difference between the subendocardial T1 (smaller in CA) and blood T1 (higher in CA) sensitivity and specificity for detection of CA compared to controls increased to 90% and 87%, respectively (50). Initial studies examining LGE in CA found this phenomenon in a majority (>70%) of CA patients (50, 54). However, the reported patterns of LGE differed possibly due to varying patient populations and technical differences in image acquisition, including in myocardial nulling. In traditional CMR imaging, “nulling” is an operator dependent process where an inversion time is selected to “null” or “make black” normal myocardium. Incorrect selection can actually reverse signals which confounds interpretation. Therefore “nulling” can be different in a diffuse myocardial process where there may be no normal myocardium. Multiple studies have noted more difficulty with this process for CA patients (54, 57). Phase-sensitive inversion recovery (PSIR) is a newer CMR sequence that negates this issue of improper nulling and can allow for more consistency. Fontana et al. showed traditional techniques using operator nulling were discordant from T1 maps the majority of the time whereas PSIR was fully concordant (58). These issues stress the importance of experienced CMR operators as well as the use of operator independent CMR protocols for accurate detection of CA.

With time and newer techniques, two predominant patterns of LGE in CA emerged including a global subendocardial pattern and a diffuse transmural pattern. These likely exist on a continuum from subendocardial to transmural enhancement as the disease progresses (59). Global subendocardial enhancement differs from subendocardial enhancement seen in aborted myocardial infarction as the latter correlates with vascular territories of the infarcted vessel. One study evaluating use of CMR for work-up of restrictive cardiomyopathy with associated LVH demonstrated a sensitivity of 80% and specificity of 94% for a global subendocardial LGE pattern compared to the gold-standard of endomyocardial biopsy in diagnosing CA (57). Similar performance in detecting cardiac involvement was seen for any LGE pattern in a group of systemic AL patients (48). Different subtypes of CA are known to demonstrate LGE patterns with varying frequencies. Dungu et al. (53) examined 100 patients with CA, with all but 1 having LGE. The “classic” subendocardial pattern was seen in 39% of AL-CA and 12% of ATTR-CA whereas a transmural involvement of affected segments occurred in 37% of AL-CA and 90% ATTR-CA and. A global LGE pattern was seen in 4% of AL-CA patients and 22% ATTR-CA (53). Similar frequencies and distributions were seen in other studies (58, 60). Additionally, a greater degree of LGE was present in ATTR-CA compared to AL-CA.

Based on this, the Query Amyloid Late Enhancement (QALE) tool, a semi-quantitative way to assess degree of LGE, was developed. In short axis, the LV is divided into three segments (base, mid, and apex) and each segment is scored from 0 to 4 (0 = no LGE, 1 = non-circumferential subendocardial OR patchy LGE, 2 = circumferential subendocardial LGE, 3 = any transmural LGE, and 4 = circumferential transmural LGE). An additional six points is added if RV LGE is present, which is more common in ATTR-CA (48, 60). A QALE ≥ 13 predicted ATTR-CA over AL-CA with a sensitivity of 82% and specificity of 76%. Combining QALE with age and interventricular septal wall thickness in a logistic probability unit increased sensitivity and specificity to 87 and 96%, respectively (53). In addition to being able to distinguish between ATTR-CA and AL-CA LGE patterns may vary between wtATTR and hATTR (48, 60).

Atrial LGE may also be a useful diagnostic marker in CA. Kwong et al. found left atrial LGE in 78% of CA patients compared to 14% of HHD patients and 9% of patients with non-ischemic cardiomyopathy (55). This study also demonstrated increased discriminatory power of having multiple LA segments with LGE in CA compared to non-amyloid HF. More so, interatrial LGE may be more common in ATTR-CA compared to AL-CA (55). While CMR can be suggestive of a specific subtype of CA, further testing is needed to definitively identify causative protein (Figure 1).

While use of Gd contrast is preferred in the CMR evaluation of CA, severe renal dysfunction, a common co-morbidity in CA, may preclude use. However, novel T1 mapping sequencing techniques that do not utilize contrast have shown to be useful in identifying CA compared to other forms of HF. In one particular study, the T1 signal was higher in a cohort of AL-CA patients compared to healthy volunteers and in patients with aortic stenosis (61). Other studies have shown consistently higher global T1 values in ATTR-CA as compared to non-CA controls (62). While global T1 values are beneficial, regional T1 variations in CA are also seen. Similar to the pattern seen with other imaging parameters, higher T1 in basilar segments compared to apical segments is commonly seen and can help differentiate CA from other disease states where T1 may be increased (63, 64). Regional areas of higher T1 correlates with lower strain in the same segment as well as extracellular volume (ECV) and LGE (63, 65). Incremental increases in basal and mid T1 correlates with higher mortality as well (64). Acquiring T1 mapping signals to detect CA is not usually standard CMR protocol so referring physicians would need to specify CMR indication and potentially discuss with the radiologist about required sequences to aid in diagnosis.

### Emerging Techniques

Quantification of myocardial ECV, a marker of myocardial tissue remodeling, is a relatively novel technique that is becoming more widespread in diagnosis and prognostication of CA. ECV is similar to T1 but the latter incorporates extracellular and intracellular factors and is heavily influenced by water content like in edema. CA is a complex interplay between amyloid fibrils, cardiomyocytes, and edema, which all affect T1 signal. Therefore, ECV is likely superior in providing a true quantification of amyloid burden (66). CA patients have consistently elevated ECV compared to healthy and other disease state controls. In a study by Kim et al. (67), the degree and relative distribution of increased ECV varied by LGE pattern. In patients with diffuse transmural LGE, the basal segments had higher ECV compared to the apex. The opposite was seen in healthy controls who tended to have a higher ECV at the apex. Conversely, patients with subendocardial or other patterns of LGE had no base-apex variation in ECV, despite having higher absolute values of ECV in all segments compared to healthy controls, likely indicating diffuse amyloid deposition but to a lesser degree than in individuals with diffuse transmural LGE (67). Finally, elevated ECV was detected in patients with a high probability of CA in the absence of LGE likely indicating early disease (66).

Strain imaging using CMR is evolving and may overcome the technical challenges of echocardiographic strain. Contrary to echocardiography, strain analysis with CMR is not reliant on imaging plane and therefore can be evaluated in multiple dimensions with greater certainty providing a more complete picture of myocardial function. Kim and colleagues (67) demonstrated worse peak strain in all dimensions (circumferential, radial, and longitudinal) in patients with CA compared to healthy controls. Additionally, a continuum of worsening strain was demonstrated from focal or patchy LGE to diffuse LGE. There was also a correlation between basal peak circumferential strain and basal ECV supporting the theory that amyloid burden is the primary driver of abnormal strain (67). These techniques also allow for strain analysis in the more complex geometry of the right ventricle.

In addition to multi-dimensional strain analysis, CMR strain techniques have higher spatial resolution than echocardiography thereby providing information on complex strain relationships, like twisting and shearing, throughout the entire thickness of the myocardium. Early CMR strain techniques used deformable registration algorithms (DRAs) that relied on the routinely obtained cine images. Newer techniques utilize changing magnetic fields and radiofrequency (RF) pulses along with time delays between generation and detection to measure deformation. Some of these create local alternations in tissue signal with magnetic fields and RF pulses creating an array over the myocardium. Through the cardiac cycle, movement of each part of the array can then be tracked and deformation parameters determined. The strain of myocardium can also be encoded in the detected signal after excitation either using stimulated echo (DENSE) or unbalanced gradient pulse during excitation and again prior to detection (strain-encoded, SENC). These latter techniques have higher spatial resolution than tagged techniques as each pixel is “encoded” with strain data instead of artificially creating a strain array (68). All of these techniques require special imaging protocols and therefore expertise in performing them. These special protocols will also add imaging time which could affect patient comfort. As advanced CMR strain techniques become more widely used in CA, the impact of specific strain patterns on symptoms and prognosis should become more evident and may eventually help guide treatment.

### Prognosis

Many morphologic and tissue characteristics seen on CMR have prognostic significance. Understanding prognosis based on these findings may inform treatment decisions while framing expectations for patients and families. It remains to be seen how pre-treatment imaging characteristics may guide prognosis in the era of contemporary therapy for CA.

Like echocardiography, static dimensions and function parameters on CMR have been shown to correlate with mortality in CA. Abnormal LVEF or RVEF generally indicates worse prognosis with degree of systolic dysfunction correlating with increased morbidity and mortality (47, 52). Lower indexed RV volumes (RVESVi or RVEDVi) were also associated with worse survival in CA and can be consistently measured with CMR (51, 52). The prognostic significance of LGE is mixed in the literature, likely related to inconsistencies in imaging protocol as well as variable follow-up lengths across studies. In general, the presence of LGE predicts a worse prognosis in CA compared to the absence of LGE (51, 58). Fontana et al. showed a clear relationship with mortality at 24 months in a large study of both AL and ATTR-CA based on LGE pattern including 92% survival in those without LGE, 81% survival with subendocardial LGE, and 61% survival with transmural LGE (51, 58, 60). Conversely, RV LGE was a consistent predictor of worse prognosis (47, 51, 52, 69). More so, higher mortality rates have been demonstrated in individuals with higher ECV, again reflecting disease burden (60). Early studies showed reduced ECV in response to treatment, suggesting this could be a marker of treatment efficacy (66, 70). Overall, the prognostic significance of LGE and ECV is not surprising as they function as surrogate markers of total amyloid burden within a progressive disease process.

## Nuclear Imaging

In medicine, nuclear imaging techniques are ubiquitous with diverse indications. The fundamental principle involves radioactive labeling of a tracer that has affinity for a specific organ or disease process. Use of nuclear imaging in CA provides certain advantages over echocardiography and CMR including: (1) diagnostic specificity for type of amyloid fibril uptake and (2) detection of early or subclinical disease which may allow for earlier treatment. Additionally, nuclear techniques, and particularly positron emission tomography (PET), in additional to CMR, incorporate semi-quantitative measurements, which may be followed for treatment response in AL-CA and the now more readily treatable ATTR-CA. The main nuclear imaging technique being used in the diagnosis of CA is cardiac scintigraphy using labeled phosphonate tracers; however, PET imaging is emerging with growing interest.

### Cardiac Scintigraphy

Phosphonate molecules labeled with a radioactive tracer, usually technetium-99m (^99*m*^Tc), combined with a nuclear detector, was originally developed for bone imaging. Increased uptake represented areas of higher bone turnover in fractures, metastases, and osteomyelitis. Affinity of phosphonate tracers for amyloid deposition in soft tissue has been recognized for more than 3 decades (71). The exact mechanism of this affinity is unclear, but is thought to result from calcium deposition within amyloid fibrils (72).

Similar to early CA research in echocardiography and CMR, initial studies examining cardiac scintigraphy in CA were limited due to a low number of patients and ill-defined patient populations that leaned heavily toward AL-CA. Additionally, despite consistent demonstration of deposition in other organs affected by amyloidosis with multiple different phosphonate tracers, cardiac uptake was inconsistent (71, 73). Despite these early limitations, with improved patient selection, the usefulness of cardiac scintigraphy for diagnosis of ATTR-CA became evident.

Three main phosphonate tracers are labeled with ^99*m*^Tc in routine clinical practice for CA: 3,3-diphosphono-1,2-propanodicarboxylic acid (DPD), hydroxymethylene diphosphonate (HMDP), and pyrophosphate (PYP). The selection of radiotracer type is generally based on availability although research continues on inter-radiotracer performance. DPD is most commonly used in Europe whereas PYP is generally used in the United States (74). HMDP is less studied than the other two radiotracers and therefore the performance characteristics are considered less refined. Cardiac or chest single-photon emitted computed tomography (SPECT) and planar images are typically obtained 1 hour after radiotracer injection. Imaging can be delayed to 3 hours if persistent blood pool activity is noted, however. Planar imaging is rapid, and allows for visual interpretation and quantification of the degree of myocardial uptake. SPECT imaging is necessary to simultaneously perform to confirm uptake is seen in the myocardium and not in the blood pool or an extra cardiac focus (75, 76).

**FIGURE 6:**
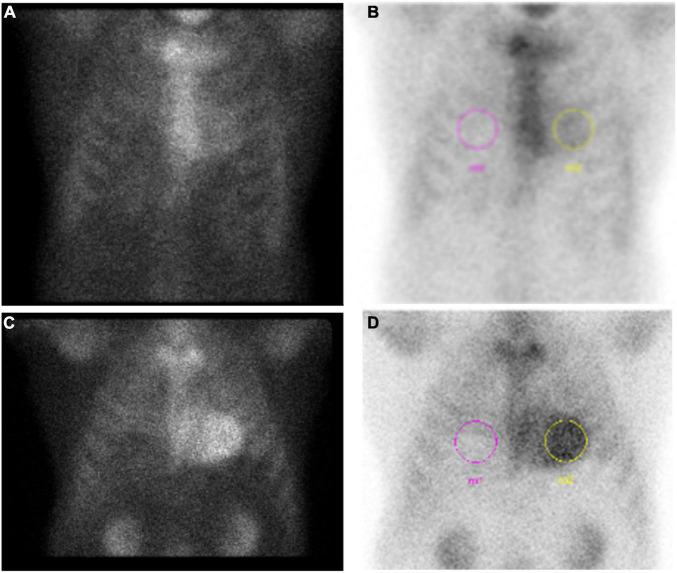
Example results of pyrophosphate (PYP) scan in two patients diagnosed with ATTR-CA. **(A)** Grade 1 (negative) scan with **(B)** H/CL = 1.4 in a patient later diagnosed by endomyocardial biopsy. **(C)** Grade 3 (positive) PYP scan with **(D)** H/CL = 2.26.

The major breakthrough in cardiac scintigraphy imaging for CA came from the hypothesis that variable uptake in earlier studies resulted from different amyloid protein composition (77). In 2005, Perugini and colleagues were the first to demonstrate this in a study of 25 patients (15 ATTR-CA) where sensitivity and specificity of 100 and 100%, respectively, was shown for distinguishing ATTR-CA from AL-CA (77). Genotyping and immunohistochemistry served as the gold standard and scan positivity was based on qualitative analysis of uptake using a scoring system that is still applied in clinical practice: grade 0 = no cardiac uptake and normal bone uptake; grade 1 = cardiac uptake intensity less than bone uptake; grade 2 = cardiac uptake intensity similar to bone uptake; and grade 3 = cardiac uptake intensity greater than bone uptake or even absent bone signal (72). In their initial study, a scan was considered positive if above a grade 0. Examples of scan results in ATTR-CA are shown in Figure 6.

To limit inter-observer variability and provide more quantitative rigor, ratios of heart to other body part uptake are also used, with the most common in modern practice being heart-to-contralateral chest ratio (H/CL) (Figures 6B,D) (78, 79). The initial study had excellent differentiation of AL-CA from ATTR-CA (78) using a cutoff of H/CL ≥ 1.5, but subsequent studies fall short on sensitivity while maintaining high specificity (79). Per current guidelines, H/CL ≥ 1.5 is positive, < 1.0 is negative, and in between is equivocal (75). Some physicians use a cutoff of H/CL ≥ 1.3 to increase sensitivity (80). Other signal ratios are scattered throughout the literature including heart to whole body (H/WBR) and heart to skull (H/S).

The high sensitivity and specificity of DPD seen in the initial study is stunning and ultimately was not nearly as stark for larger DPD studies or the other radiotracers. In other studies, a subset of patients with AL-CA ranging from 32% to 41%, had myocardial uptake with DPD on cardiac scintigraphy (81–83). With PYP, one study showed 17% of AL-CA patients had grade ≥ 2 uptake (78). HMDP use may lead to less uptake for AL-CA, but this is at the cost of lower sensitivity for diagnosis of ATTR-CA (72, 83). Larger studies pooling the main radiotracers demonstrate that ≥grade 1 on bone scintigraphy has a sensitivity > 99% and specificity 68% for diagnosis of ATTR-CA (74). Using grade 2/3 increases specificity to 87% at the cost of lower sensitivity to 91% (74). A meta-analysis pooling 529 patients across 6 studies found similar sensitivity and specificity of 92% and 95%, respectively (72). All patients in the large study by Gillmore et al. (74) underwent appropriate screening for AL-CA, but the methods in each study included in the meta-analysis are less clear. Treglia et al. also pooled the cohorts based on radiotracer used with grade 2/3 considered positive: DPD (sensitivity 95%, specificity 88%), PYP (sensitivity 87%, specificity 75%), HMDP (sensitivity 86%, specificity 98%) (72). In current clinical practice, grade 2/3 is considered positive, grade 0 is negative and grade 1 is equivocal and needs to be interpreted within the specific clinical context (Figure 6A).

Fortunately, the diagnostic accuracy of cardiac scintigraphy can be greatly enhanced with the addition of appropriate serologic work-up to rule out systemic AL amyloidosis. This includes serum free light chain measurement and ratio along with serum and urine electrophoresis with immunofixation. If these tests are normal, abnormal cardiac scintigraphy with grade of 2 or 3 uptake has a specificity of 100% for ATTR-CA (74). This stresses the importance of appropriate serologic work-up before pursuing and certainly before interpreting cardiac scintigraphy for possible ATTR-CA (Figure 1).

While specificity in this scenario approaches 100%, the sensitivity is <100%, highlighting the possibility of missing a diagnosis of ATTR-CA. Reasons why certain patients may have a false negative scan are under active investigation. One obvious reason is the increased recognition of CA leading to earlier diagnostic work-up. Degree of amyloid deposition along with clinical and biomarker characteristics correlate with Perugini grade (74, 83). These characteristics also correlate with degree of amyloid deposition on CMR (58). The initial use in more advanced disease led to high sensitivity and specificity but more contemporary ordering practices aimed at early diagnosis may have less uptake which may not be detectable below a certain amount of total amyloid deposition (74, 80–82). However, the lower sensitivity is not entirely explained by early disease as some patients with advanced disease by symptoms and other imaging have grade 0/1 uptake (83, 84).

There is building evidence that different ATTR genetic variants may lead to more or less radiotracer uptake. Therefore, some variants may lead to grade 0/1 uptake despite a heavy burden of disease. Musumeci and colleagues (84) retrospectively examined 19 DPD and HMDP scans over nearly 2 decades in hATTR patients with a Phe64Leu variant. Seventeen (85%) of these patients had grade 0/1 uptake (84). Alternatively, patients with Val122Ile, in a different study had grade 3 uptake (83). Ser77Tyr also seems to have reduced frequency of high-grade uptake but the data are limited (60). Genotyping is often performed after clinical and imaging diagnosis so a “negative” cardiac scintigraphy is not likely to trigger genetic testing (85). It is incumbent on the physician to pursue additional testing in those patients whom clinical suspicion remains even if cardiac scintigraphy is negative or equivocal.

Alternatively, some patients without suspicion of CA may undergoing cardiac scintigraphy for another indication and be found to have positive myocardial uptake. Two recent studies have examined the frequency of grade 2/3 cardiac uptake on patient scans performed for indications other than evaluation of CA. Soumalainen et al. (80) looked at 2,000 patients undergoing HMDP scans mostly for prostate and breast cancer. They found grade 1 in 16.7%, grade 2 in 2.7%, and grade 3 in 0.8% of this cohort. Additionally, 2.4% of patients had an H/CL ≥ 1.3 suggestive of ATTR-CA. Notably, of those with suspected ATTR-CA (grade 2/3), 41% had a history of HF prior to scan (80). Bianco et al. found a prevalence of 0.54% grade 2/3 uptake on 3,228 scans performed over 5 years using DPD or HDMP at their center (86). With higher clinical suspicion, ATTR-CA may have been suspected in some of these patients as 48% had prior HF, 34.8% had known neuropathy, and 21.7% had carpal tunnel syndrome. However, 21.7% were entirely asymptomatic highlighting the existence of an asymptomatic disease state during which treatment could prevent disease progression (86).

### Prognosis

The prognostic significance of cardiac scintigraphy ordered for assessment of CA is limited by the qualitative nature of the grading system although semi-quantitative results provide more granularity. Using semi-quantitative analysis, generally higher heart to other structure ratios is associated with a worse prognosis. Galat and colleagues found a H/S (heart to skull) ratio ≥ 1.94 had a higher chance of major cardiovascular event (MACE) along with NYHA III or IV symptoms (83). Another early study found that H/WB (heart to whole body) ratio > 7.5 was associated with worse prognosis (81).

For incidentally discovered myocardial uptake, the presence of myocardial uptake still portends a worse prognosis. In one study, 51% of patients died over a mean 4 years of follow-up, 9% of which were classified as related to a cardiac cause. Grade 3 uptake and H/CL ≥ 1.3 were predictors of mortality on univariate analysis and grade 3 uptake remained a mortality predictor on multivariate analysis (80). These patients likely had undiagnosed ATTR-CA which progressed in follow-up ultimately leading to death. If myocardial uptake is incidentally found, complete work-up including serologic screening for AL disease and genetic testing should be facilitated.

Currently there is no known utility of repeating scans for assessment of disease burden, but this is not yet studied in the current era of ATTR-CA treatment. One potential scenario for a repeat scan may be if a prior scan was negative despite a known ATTR genotype positivity and a repeat scan is done after some interval to assess for CA; though the optimal time frame is unknown and likely depends on the age of the patient as well as specific family history (81).

### Positron Emission Tomography

Positron emission tomography (PET) imaging has many established cardiovascular disease indications, but use in CA is emerging. Like cardiac scintigraphy, the goal of PET is to have high fidelity to identify the type of amyloid fibril protein but also to have a more quantitative method to assess amyloid burden and response to treatment. Prior to determining its role in the latter, optimally performing radiotracers for AL and ATTR-CA amyloidosis need to be identified.

Multiple pilot studies using different radiotracers have been studied using either fluorine-18 (^18^F) or carbon-11 (^11^C), with ^18^F being more common (87–90). Fludeoxyglucose (FDG) is a commonly encountered compound in oncology but its use in CA is not established (91). In contrast to bone scintigraphy, some PET radiotracers like ^18^F-florbetaben may have more affinity for AL-CA compared to ATTR-CA. Myocardial tracer retention (MTR) is calculated using standardized uptake value (SUV) in the early time frame (0–5 mins) and a delayed time frame (15–20 mins) then expressed as a percentage. In a small study inclusive of 22 patients with CA, eight patients with AL-CA had a median MTR of 66% compared to 42% in the five patients with ATTR-CA (p < 0.01). In non-CA patients, median MTR was 27% (91). A sensitivity and specificity of 100% and 100%, respectively, for ruling out amyloidosis was achieved with MTR ≤ 36%. An MTR cutoff ≤ 52% could differentiate ATTR-CA from AL-CA with a sensitivity of 100% and specificity of 89% (91). Another study by Genovesi et al. (92) performed dynamic and static imaging at various time points after injection. All patients (AL, ATTR, other) had early uptake but AL-CA patients had a higher degree of early uptake. AL-CA patients then had much more retention of radiotracer throughout the imaging time compared to ATTR-CA which washed out much quicker (92). Larger confirmatory studies are needed but PET imaging has the potential to serve as the first non-invasive modality of diagnosing AL-CA and may decrease the need for biopsy in some patients.

Simultaneous imaging of patients using PET and CMR has also been under investigation in CA. Albuizi and colleagues used PET and CMR technology in 27 patients with strong suspicion of CA using ^18^F-NaF after a promising proof of concept study. Though qualitative measures were unreliable to distinguish CA subtypes, with semi-quantification comparing the myocardium to blood pool (M/B ratio), there was increased relative uptake in ATTR-CA patients compared to AL-CA and non-CA patients. An M/B ≥ 0.90 was able to differentiate ATTR from AL-CA with a sensitivity and specificity of 81% and 100%, respectively (93). The simultaneous use of PET and CMR could allow for identification of morphologic and tissue characteristic changes supporting the presence of CA, then utilizing PET to identify the CA subtype.

PET results have been shown to correlate to findings on other imaging modalities including echocardiography, CMR, and cardiac scintigraphy. MTR had a positive correlation with apical sparing, E/e′, and wall thickness along with a negative correlation with TAPSE and LVEDV on echocardiography (91). PET had 94% concordance regarding extent of affected myocardium on CMR. Interestingly, in one patient, a positive PET study did not have any CMR abnormalities, which may point to a role in earlier diagnosis. Cardiac scintigraphy (DPD) matched PET results in 81% of patients. Notably all ATTR-CA patients in this study had grade 3 uptake (91).

Follow-up PET scans do not have a defined role in clinical practice, but this remains a promising avenue of future study. In the study by Kircher et al. (91), four patients had repeat PETs either for treatment follow-up or restaging. In all cases, imaging findings correlated with clinical status. One patient had a repeat scan after approximately 1 year of treatment with ATTR stabilizer, Tafamidis, showing stable MTR and correlating with stable HF symptoms as well. One patient had improvement in biomarkers but worsening clinical status which correlated with a higher MTR on follow-up scan; this patient went on to receive a heart transplant (91). Repeat PET scans may serve as a way to assess response to treatment and decide if escalation or alternatives are needed. The main limitation to PET in this context will be cost, availability, and radiation exposure. As experience accumulates, the impact of genetic variant on radiotracer uptake will also need to be investigated given the emerging data seen with cardiac scintigraphy. Finally, the prognostic implications of PET imaging in CA have not been investigated beyond anecdotal reports.

## Role of Endomyocardial Biopsy in Cardiac Amyloidosis Diagnosis

Once a clinical suspicion of CA is established, further testing focuses on culprit protein identification. All patients need a serologic assessment of AL amyloidosis consisting of serum light chain measurement and ratio (kappa/lambda ratio), and urine and serum electrophoresis with immunofixation (Figure 1). If any of these tests are abnormal, AL amyloidosis must be expeditiously evaluated with tissue biopsy and referral to hematologic oncologist. An abnormal kappa/lambda ratio (>1.65–3.1) in the absence of a monoclonal gammopathy on protein electrophoresis may create a diagnostic dilemma in those with renal dysfunction, as the kidneys are responsible for clearing light chains. In this setting, clinical correlation is recommended to determine suspicion for AL amyloidosis, which can then guide further diagnostic testing (76). If serologic testing for AL amyloidosis is all within normal limits, AL-CA is effectively ruled out and cardiac scintigraphy can be performed to assess for ATTR-CA (Figure 1). If this is positive (grade 2/3), a diagnosis of ATTR-CA can be made without further testing. If cardiac scintigraphy is negative or equivocal, echocardiography, CMR, and patient history needs to be reviewed and referral for tissue biopsy should be considered for patients with ongoing clinical suspicion for CA. While rare, endomyocardial biopsy may also detect dual pathology of AL and ATTR-CA, further highlighting the importance of pursuing biopsy in the appropriate clinical context.

**FIGURE 7:**
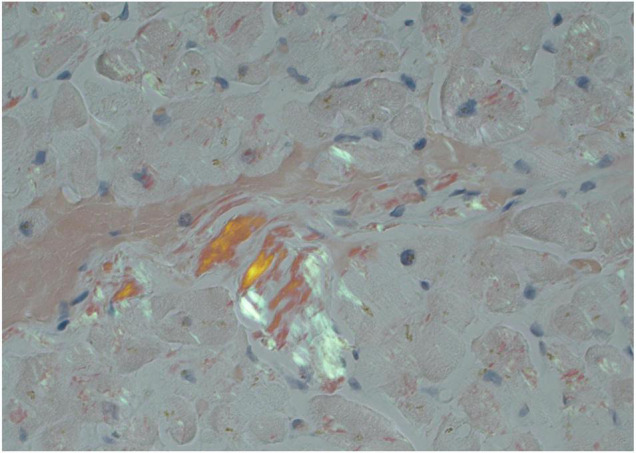
Endomyocardial biopsy pathology in a patient with cardiac amyloidosis. Congo red stain at 10×x magnification showing apple-green birefringence under polarized light consistent with amyloid deposition.

The site chosen for tissue biopsy varies by center. Abdominal fat pad biopsy may be first pursued as this is a minimally invasive and well-tolerated procedure. However, sensitivity is very poor for ATTR-CA and ranges considerably for AL-CA (94–96). Therefore, if negative, endomyocardial biopsy ought to be pursued for definitive diagnosis. In modern practice, most endomyocardial biopsies are performed on the RV septum through venous access. The major complication of RV biopsy is cardiac perforation leading to tamponade. Minor complications of biopsy include ventricular arrhythmias, access site bleeding, arterial injury, pericardial effusion, and conduction disturbances. Major complications occur on <1% of RV biopsies and minor complications occur in 2–5% of cases (97). CA is a diffuse process so diagnostic yield is greater than patchier myocardial processes and with adequate samples, the sensitivity and specificity approach 100% and 100%, respectively. In very early disease, amyloid deposition may be patchy thereby decreasing yield but these situations are likely rare. CA will classically have the appearance of Congo red staining on histology, with an apple-green birefringence appearance under polarized light (Figure 7). Once amyloidosis is identified on tissue biopsy, the tissue should be then be typed with either mass spectrometry or immunohistochemistry to further determine subtype (96).

All patients diagnosed with ATTR-CA, whether non-invasively or with biopsy, must undergo genetic testing to establish a diagnosis of either hATTR or wtATTR. Genetic testing is typically performed with salivary kit testing and in conjunction with genetic counseling. Identification of hATTR can trigger cascade screening in family members.

## Conclusion

CA is underdiagnosed but improved treatment options have created a diagnostic imperative for earlier and more widespread recognition. Non-invasive evaluation is the backbone of this process and multiple imaging modalities are integral to this process including echocardiography, CMR, and nuclear techniques. CA must first be recognized based on clinical clues and supportive echocardiogram or CMR features. Once CA is suspected based on these imaging modalities, serologic markers, nuclear imaging, and in some cases tissue biopsy allow for on culprit protein identification. Specificity for subtype of CA is high using this algorithm but sensitivity falls short for multiple reasons still being investigated. Endomyocardial biopsy continues to play a pivotal role in CA work-up for a subset of patients in whom CA subtype is unclear or high suspicion remains despite negative or equivocal serologic and imaging work-up. Recognizing when biopsy should be pursued can decrease treatment delays and ultimately increase life expectancy in CA patients.

## Author Contributions

PS and JV conceptualized the content of the article, drafted the manuscript, and obtained appropriate images. MM and AH conceptualized the content of the article, provided extensive critical review, and edited the manuscript. All authors contributed to the article and approved the submitted version.

## Conflict of Interest

The authors declare that the research was conducted in the absence of any commercial or financial relationships that could be construed as a potential conflict of interest.

## Publisher’s Note

All claims expressed in this article are solely those of the authors and do not necessarily represent those of their affiliated organizations, or those of the publisher, the editors and the reviewers. Any product that may be evaluated in this article, or claim that may be made by its manufacturer, is not guaranteed or endorsed by the publisher.
